# Practical Implications of Empirically Studying Moral Decision-Making

**DOI:** 10.3389/fnins.2012.00094

**Published:** 2012-07-06

**Authors:** Nora Heinzelmann, Giuseppe Ugazio, Philippe N. Tobler

**Affiliations:** ^1^Faculty of Philosophy, University of OxfordOxford, UK; ^2^Laboratory for Social and Neural Systems Research, Department of Economics, University of ZurichZurich, Switzerland

**Keywords:** descriptive, morality, normative, reasoning, neuroethics

## Abstract

This paper considers the practical question of why people do not behave in the way they ought to behave. This question is a practical one, reaching both into the normative and descriptive domains of morality. That is, it concerns moral norms as well as empirical facts. We argue that two main problems usually keep us form acting and judging in a morally decent way: firstly, we make mistakes in moral reasoning. Secondly, even when we know how to act and judge, we still fail to meet the requirements due to personal weaknesses. This discussion naturally leads us to another question: can we narrow the gap between what people are morally required to do and what they actually do? We discuss findings from neuroscience, economics, and psychology, considering how we might bring our moral behavior better in line with moral theory. Potentially fruitful means include nudging, training, pharmacological enhancement, and brain stimulation. We conclude by raising the question of whether such methods could and should be implemented.

## Introduction

A sharp distinction has been made between the descriptive domain of morality, i.e., the way agents behave or make moral judgments, and the normative domain, i.e., the way agents ought to behave or make moral judgments. In the empirical sciences, there has been an on-going debate about which theory describes moral decision-making best. Similarly, normative moral philosophy has been discussing which ethical theory is superior to the others.

However, whenever we watch the news or observe our social environment, both of these issues are of comparably little importance to us. The question that usually concerns us is not: How do people behave? Or: How ought they to behave? But rather: Why do they fail to behave in the way they should?

This last question is not purely an empirical one, as it involves an assumption about how one ought to behave. Nonetheless, it is neither an ultimately normative one, as it relies on empirically observable facts about human behavior. The issue is rather a practical one, reaching both into the descriptive and the normative domains of morality. It naturally leads to another practical question: What can we do about the fact that people often do not behave in a way they are morally required to?

In this essay, we elaborate on these two related practical issues and give an outline of how to resolve them. We argue that two main problems usually keep us from acting and judging in a morally decent way: Firstly, we make mistakes in moral reasoning. Secondly, even when we know how we ought to act and judge, we still fail to meet our obligations due to personal weaknesses.

## How Ought We to Act?

Normative ethics tells us what we ought to do. Three of the most prominent contemporary theories are consequentialism, deontology, and virtue ethics (Crisp, 1998/2011, cf. Tobler et al., 2008). There is no clear, simple, and universally accepted definition for any of them; therefore we shall give a brief account of how these concepts are understood in the present paper. Albeit rough and sketchy, we assume that these characterizations serve our present purpose well enough.

In one of its general forms, consequentialism tells us that the outcomes (consequences) of our actions ought to be as good as possible (cf. Scheffler, 1988). There are numerous consequentialist theories which in turn can be classified in various ways. Philosophers traditionally distinguish act and rule consequentialism. Act consequentialism holds that the outcome of single actions ought to be as good as possible. As consequences of single actions are often difficult to predict, attempts have been made to facilitate the decision process of an agent. In this vein, rule consequentialism focuses on action-guiding rules, claiming that the consequences of the rules be as good as possible. Actions are then evaluated with respect to these rules.

Also, different consequentialist approaches disagree on what the goodness of an outcome consists of. The most popular one, utilitarianism, holds that we ought to do what increases people’s happiness or decreases their unhappiness. Hereby, the good of everyone has to be taken into account and everyone’s good counts equally. We ought to act in a way that maximizes the good of all and in no other way. Jeremy Bentham, one of the founders of classical utilitarianism, argued for a felicific calculus that allows measuring the outcome of various actions, i.e., the pleasure these actions may produce. Such a method presupposes that all pleasures are comparable and quantifiable and that they are, as consequences of an action, to greater or lesser certainty predictable. After such hedonic approaches to (experienced) utility had been largely abandoned by economics, they have more recently been taken up again by behavioral economics (Kahneman et al., 1997). Moreover, some formal treatments of welfare economics (Harsanyi, 1955) and prosocial preferences (e.g., Fehr and Schmidt, 1999) also have consequentialist roots.

“Deontology” is a collective term denoting a variety of theories which, from a linguistic point of view, assign a special role to duties, as “deontology” refers to the study or science of duty (deon = duty). Deontology requires us to fulfill our moral duties but such a general claim is also made by consequentialist theories, which hold that it is our moral duty to act in such a way that the outcomes be as good as possible. Therefore, deontology is sometimes identified with non-consequentialism, the claim that the wrongness or rightness of an action is not only determined by the badness or goodness of its consequences. For instance, an action can be assigned intrinsic value because of the agent’s willingness that the principle – or maxim – on which the action is performed should become a universal law, a criterion established by Kant (1965/1785). Kant’s ethics and the theories derived from them are often seen as prominent candidates of deontology. Another central requirement of Kant’s ethics is to never treat a human being as a means to an end. Thus according to Kant and in contrast to consequentialism, it would be morally wrong to kill one person if thereby two other human lives could be saved.

Usually, deontology is schematically conceived of as rivalling both consequentialism and virtue ethics. Virtue ethics usually goes beyond the question of what we morally ought to do. This has historical reasons: The earliest prominent account of virtue ethics has been developed by Aristotle (2000) who was concerned with the best way for a human being to live. A central claim of contemporary virtue ethicists is that living virtuously is required in order to flourish. Roughly speaking, a virtue is a disposition to act appropriately for the right reason and thus requires practical wisdom. Flourishing can be described as living fulfilled and happily, which goes beyond mere momentary subjective well-being but refers to an overall outlook and life as a whole.

All of these theories are primarily concerned with the question of how we ought to act rather than how individuals actually do behave. We shall turn to this topic in the following section.

## How Do We Act?

Empirical research on human moral behavior has focused primarily on two topics: action and judgment. As these two aspects of moral behavior have been studied using rather different approaches, we shall treat each of them separately here. First, we consider the literature studying the effects of norms on people’s actions (Bicchieri, 2006; Gibson et al., in press). Second, we shall focus on the literature studying the psychological mechanisms underlying moral judgments (Greene et al., 2001; Moll et al., 2005; Hauser, 2006; Prinz, 2006; Mikhail, 2007).

From a wider perspective, the question arises whether moral judgment translates into moral behavior. This issue is controversial and has received a variety of answers (e.g., Schlaefli et al., 1985). One view (Bebeau et al., 1999) suggests that a moral act requires not only that an agent judges one course of action as moral but also that she identifies a situation as moral (e.g., that consequences of distinct courses of action have differential welfare implications; moral sensitivity), chooses the moral over other courses of action (moral motivation) and persists to implement the goal of the action (moral character). In this view, it would be expected that judgment and action are positively but weakly correlated, which seems to be the case (Blasi, 1980).

### Moral action

One of the most successful approaches to study moral action has been to observe how people’s behavior changes depending on the saliency of a norm. Scholars working in this field developed several models to show how the utility assigned by a person to different outcomes in a given situation is modified by the presence of a norm. Norms motivate compliant behavior mainly in two ways: (a) they modify the expectations an individual has regarding others’ behavior (Bicchieri, 2006) and (b) they generate a personal cost for violating the action course prescribed by the norm (Gibson et al., in press).

While Bicchieri’s work focused mainly on providing a theoretical description of how and when social norms are most likely to emerge and influence individuals behavior, other scholars provided empirical evidence demonstrating the influence of norms on behaviors in a social context. For instance, recently Gibson et al. (in press) tested the influence of the moral obligation of being honest (or not lying) on individuals’ behavior in an economic context. The authors tested the hypothesis that when being incentivized to lie by being able to make a greater profit through not telling the truth, the willingness of an individual to behave immorally, i.e., to lie, was correlated with the importance she assigned to being honest. More specifically, those individuals attributing high importance to the honesty norm were extremely insensitive to the cost of telling the truth, which suggests that the moral value of respecting a moral duty (of being honest) can outweigh economic costs of respecting it and even prevent utilitarian cost-benefit trade-offs altogether.

### Moral judgment

Whereas psychological research on moral judgments has captured them predominantly as a cognitive, controlled process, and focused on moral development in the 20th century (Piaget, 1932; Kohlberg, 1976), it has in recent years mainly developed around two research questions: (a) do moral judgments stem from intuitions or from conscious reasoning and (b) which psychological processes are involved in moral intuitions (Cushman et al., 2010). Roughly, we can distinguish four different approaches to these questions.

From a first perspective, following Hume’s (1960) idea that moral judgments result from “gut feelings”, some scholars proposed that moral judgments predominantly result from intuitions of an emotional nature (Prinz, 2006, see also Prinz, 2007; Woodward and Allman, 2007).

Second, others agree that moral judgments indeed stem from intuitions but they deny that such intuitions are of emotional nature, arguing instead that moral intuitions are the product of moral specific psychological mechanisms named “universal moral grammar” (Hauser, 2006; Mikhail, 2007; Huebner et al., 2008). According to this view, neither conscious reasoning nor emotions play a causal role in determining moral judgments, suggesting that these two processes actually occur after the moral judgment has been produced by the “moral grammar” mechanism.

From a third point of view other scholars put forward a dual-process theory of moral judgment (Greene et al., 2004) suggesting that moral judgments result from two psychological mechanisms: emotions and conscious reasoning. It is consequently claimed that different moral judgments are underpinned by different psychological systems (Cushman et al., 2010).

Finally on a very similar stance, a fourth theory acknowledges that moral judgments rely on multiple psychological mechanisms, and therefore that both emotions and conscious reasoning play a role in moral judgments. However, in contrast with the third view described above, it is argued that different moral judgments are not underpinned by different psychological systems, but rather that all moral judgments will involve cognitive and emotional mechanisms in competition against each other when a moral judgment is produced (Moll et al., 2005, 2008).

### Neural underpinnings

**Figure 1:**
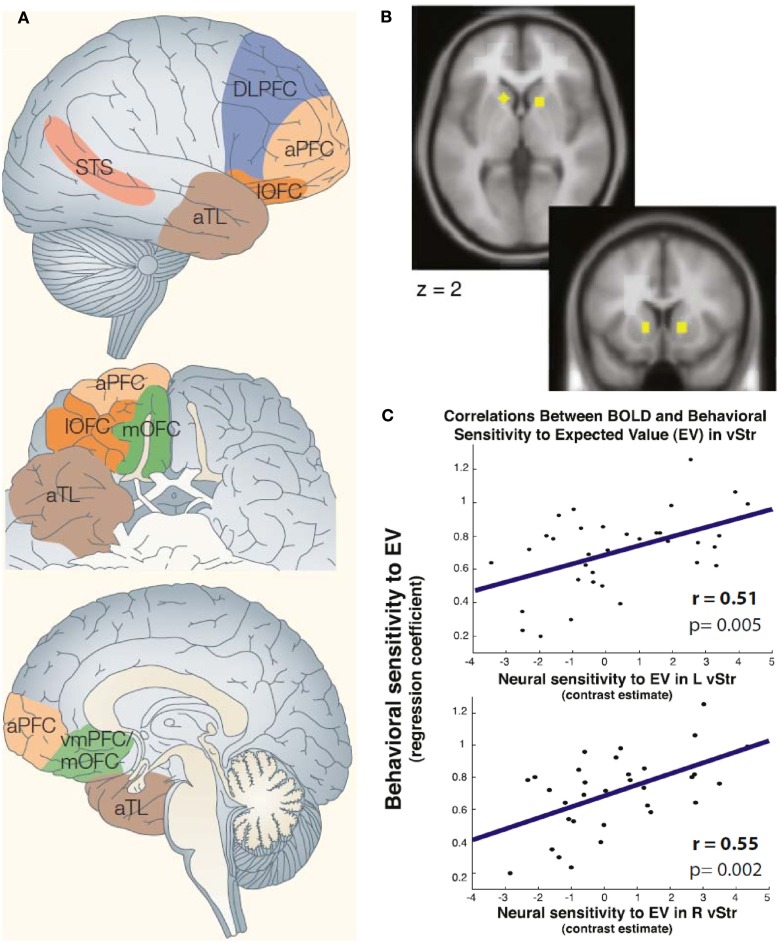
**Brain regions implicated in moral judgment and decision-making**. **(A)** Cortical regions. Note that the posterior cingulate cortex and the angular gyrus (temporoparietal junction) have also been implicated in moral judgments (shown in Figure 2). aPFC, anterior prefrontal cortex; aTL, anterior temporal lobe; DLPFC, dorsolateral prefrontal cortex; lOFC, lateral orbitofrontal cortex; STS, superior temporal sulcus; vmPFC, ventromedial prefrontal cortex. Adapted with permission from Moll et al. (2005). **(B,C)** Example for striatal involvement in moral decision-making. The task employed moral dilemmas. In each trial, subjects rated how morally acceptable it was to save a group of individuals from death with a known probability rather than a single individual with certainty. Across trials, group size, and probability varied. Group size and probability should be multiplied to compute the expected number of lives saved. **(B)** Regions in ventral striatum previously identified by Knutson et al. (2005) as processing reward value. **(C)** In the regions shown in **(B)**, individual neural sensitivity (contrast estimates of activation increases) correlated with behavioral sensitivity (beta estimates in rating) to the expected number of lives saved. Adapted with permission from Shenhav and Greene (2010). This finding is in line with the notion that moral functions can be underpinned by neural mechanisms that have originally evolved for different functions, such as reward processing (Tobler et al., 2008).

**Figure 2:**
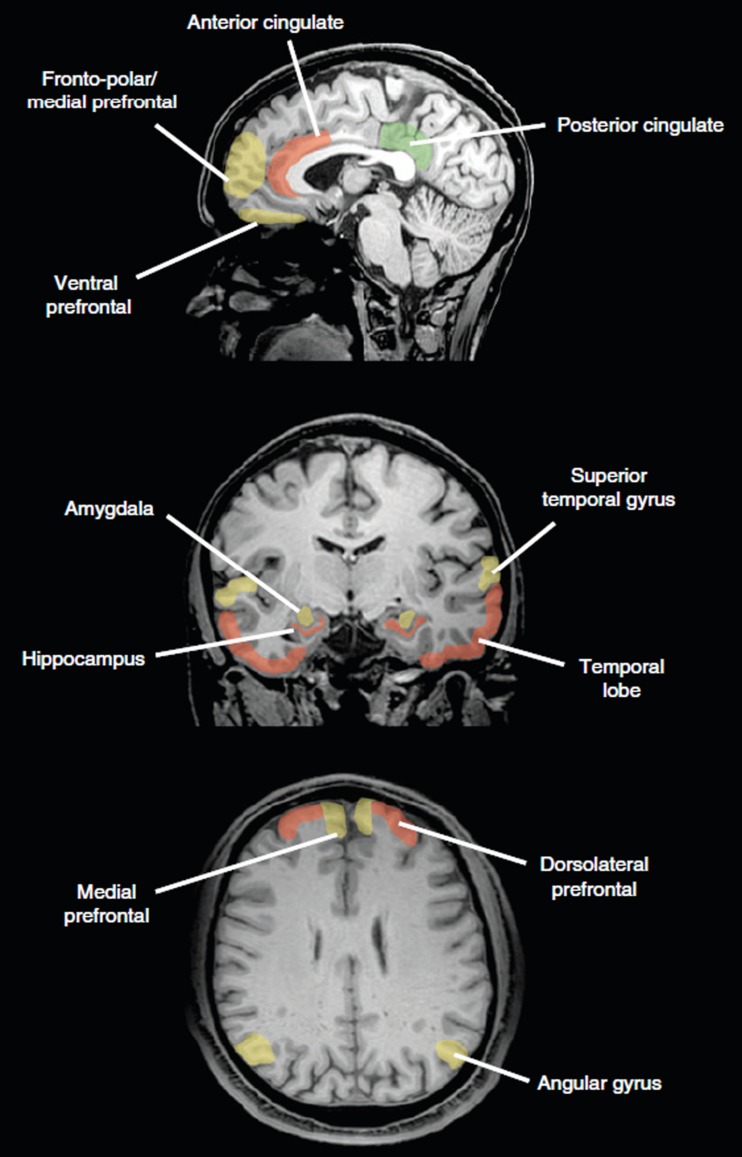
**Comparison of brain regions preferentially activated during moral judgment and decision-making (green), regions impaired in patients with antisocial disorders such as antisocial personality disorder and psychopathy (red) and common regions (yellow)**. One possible interpretation is that emotions as underpinned by the common regions prevent breaking of moral rules, the defining deficit of antisocial personality disorders. The angular gyrus lies at the junction of temporal and parietal cortex. Reprinted with permission from Raine and Yang (2006).

The advent of neuroimaging methods allowed to study the intact brain of healthy volunteers while they make moral judgments and decisions. This line of research has identified a variety of brain regions that are active during moral cognition (Figure 1; for review, see e.g.: Moll et al., 2005, 2008; Raine and Yang, 2006; Forbes and Grafman, 2010). These regions include the prefrontal cortex, particularly ventral, medial, dorsolateral, and frontopolar subregions, posterior cingulate cortex, anterior temporal lobe, superior temporal cortex, temporoparietal junction, striatum, insula, and amygdala. Many of these regions are also implicated in “theory of mind” tasks requiring consideration and inference of others’ thoughts and desires (Bzdok et al., 2012) and impaired in patients with antisocial disorders, in agreement with the notion of impaired moral decision-making (Figure 2; Raine and Yang, 2006).

One could next ask whether neuroimaging can contribute to informing theories of moral decision-making. Could it help deciding between the different theories outlined in 2.2 (even though some of them may not be mutually exclusive)? Or, more specifically, can neuroimaging inform us about the degree to which emotions are involved in moral judgment? When asking such questions one is often tempted to make reverse inferences from brain activation to mental function. However, given that most brain regions contribute to more than one function, such inferences are at best probabilistic (Poldrack, 2006, 2011). Moreover, they are limited by the response specificity of the brain region under study and by the precision with which mental functions are parsed conceptually and assessed empirically (Poldrack, 2006). Nevertheless, some attempts to answer those questions have been made.

For example, an extension to Hume’s view mentioned above may be suggested by the involvement of dorsal and lateral frontal regions in moral judgment (e.g., Greene et al., 2001). This would be based on the notion that these regions play a stronger role in more deliberate, goal-directed, and cognitive than automatic and emotional functions (Forbes and Grafman, 2010). Moreover, all of the regions implicated in moral judgment have been implicated also in other mental functions. This seeming lack of evidence for a neural substrate exclusively devoted to moral functions (Young and Dungan, 2012) does not support the universal moral grammar approach; if one assumes that moral functions have evolved from non-moral functions or that the mental functions required for other types of judgments can be used also in the moral domain (Tobler et al., 2008) it is perhaps not surprising that so far no region has been singled out as a uniquely moral center of the brain. In principle though it is still conceivable that finer grained methods, such as single cell recordings, may reveal such a substrate.

Neuroimaging and lesion work also point toward a role for emotion in moral judgment. The ventromedial prefrontal cortex (vmPFC) is involved in emotion processing and also activated when a subject makes moral judgments (reviewed in Young and Koenigs, 2007). Lesions of this region result in blunted affect (hypo-emotionality) as well as increased emotional reactivity to environmental events (Anderson et al., 2006). Activations are increased by pictures with moral emotive content (depicting, e.g., abandoned children, physical assaults) compared to pictures with non-moral emotive content of similar emotional valence and sociality (Moll et al., 2002; Harenski and Hamann, 2006) and by moral compared to semantic judgments (Heekeren et al., 2003, 2005). Patients with lesions of the vmPFC are more likely than controls to endorse harming someone in order to benefit a greater number of other people (Ciaramelli et al., 2007; Koenigs et al., 2007; Thomas et al., 2011). In healthy subjects the strength of skin conductance responses to such moral dilemmas correlates inversely with the propensity to endorse harm for the greater good (Moretto et al., 2010). By contrast, vmPFC patients fail to generate such emotive responses before endorsing harm (Moretto et al., 2010). Thus, at least some moral judgments appear to be caused by emotions.

Although much of the literature has focused on prefrontal cortical regions, moral judgment, and decision-making are clearly not a purely prefrontal or, more generally, neocortical matter. Activation in the striatum, for example, is affected by the moral status of a partner with whom one performs economic exchanges (Delgado et al., 2005) and reflects behavioral sensitivity to the “moral expected value” (number of lives saved) of moral actions (Shenhav and Greene, 2010; Figure 1B). Based on its general role in action selection (Balleine et al., 2009), one would also expect the dorsal striatum to contribute to the selection of moral actions. The amygdala contributes to the learning of fear and distress experienced by others (Blair, 2007; Olsson et al., 2007); empathy-induced insula activation correlates with subsequent prosocial behavior (Masten et al., 2011). Thus, although these regions may primarily serve different functions they can nevertheless be harnessed for moral judgments and decisions.

## People Do Not Behave in a Way They Ought to

Combining insights from the two previous sections, this part of the paper will establish the claim that human beings often do not behave in a way they ought to. Although it is clear that discrepancies can arise from a variety of issues, including moral sensitivity, judgment, motivation, and character, we will concentrate on two more recently discussed phenomena: cognitive biases and emotional influences.

Both these phenomena are morally problematic in that they reflect the influence of morally irrelevant features on actions and judgments. We shall briefly clarify this point for each of the three ethical theories outlined in the section “How Ought We to Act?” above.

As mentioned before, consequentialism requires that only the ultimate consequences of an action or judgment are relevant to its moral evaluation. Therefore, features such as the emotional state of the agent or the framing of several options to choose from are not to be taken into account. However, as we shall elaborate in the following, there are a variety of instances in which agents are influenced by such cues and therefore do not act and judge in a morally decent way.

From a Kantian point of view, a morally right action or judgment is to be made from duty, that is, out of reverence for the moral law. Accordingly, any other feature of a situation, such as the agent’s uneasy feeling toward the morally prescribed action course, is to be ignored. However, empirical evidence will be given below that individuals often fail to meet this normative requirement.

Virtue ethics outlines the character traits which distinguish a virtuous person. Amongst them are the faculty of practical reasoning and specific virtues such as justice or temperance. There is, however, solid evidence that agents frequently fail to display these traits in their behavior and judgments, as this section shall make clear.

In the following, we shall explain in greater detail in what ways individuals are biased or influenced by their emotions. For some cases, we shall, by way of example, explain how the actions and judgments in question are morally dubious from a deontological, consequentialist, or virtue ethical perspective.

### Biased behavior

Briefly, a cognitive bias is an unconscious tendency to judge a certain element in a way that depends on one’s own preferences, expectations, and experiences. Cognitive biases are similar to perceptual biases such as optical illusions (e.g., the Müller-Lyer illusion, Müller-Lyer, 1889). Instead of influencing our perceptual skills, cognitive biases affect people’s cognitive capacities. We shall give some examples for this phenomenon below.

Firstly, a known cognitive bias that strongly affects moral actions is the so-called *bystander effect*, i.e., “the more bystanders to an emergency, the less likely, or the more slowly, any one bystander will intervene to provide aid” (Darley and Latané, 1968, p. 1). Darley and Latané (1968) recreated an emergency situation in the lab in order to test the reactions of participants. The higher the number of bystanders, the lower the percentage of participants who decided to intervene and the longer the time it took them to do so. Presumably, people recognize the badness of the situation, yet feel a “diffusion of responsibility” and so do not act accordingly. However, such a behavior is morally questionable. For instance, from a deontological perspective, it is highly plausible to assume that an agent has a strong duty to help a victim in an emergency. Besides, such a duty is often legally prescribed, i.e., non-assistance of a person in danger is widely regarded as tort. The presence of bystanders and their number does not relieve the agent from his moral duty. Failure to act in accordance with the duty to help is thus a severe moral transgression from a deontological point of view.

Secondly, the next cognitive bias taken into consideration here is known as the *identifiable victim effect* (Schelling, 1968; Redelmeier and Tversky, 1990): one is more likely to help a victim if he is easily identifiable. An example of this behavior is people’s widespread inclination to save one little child from drowning in a shallow pond but to refrain from making a small donation that would save 25 children from starving to death in Africa (cf. Hauser, 2006). This pattern of results was consistently found in numerous previous studies observing people’s behavior in similar situations (Calabresi and Bobbitt, 1978; Redelmeier and Tversky, 1990; Viscusi, 1992; Whipple and Swords, 1992). Again, this is morally dubious behavior, as we shall argue from a virtue ethicist’s viewpoint. Generally, charity and justice (or fairness) are regarded as moral virtues. Assume further, plausibly enough, that the overwhelmingly important point about being charitable is the benefit of the person receiving aid. Then a virtuous agent would help both the drowning child and the starving kids. Helping one but not the others seems to amount to a failure of exhibiting charity and justice and therefore to non-virtuous behavior. From a consequentalist perspective it could be argued that saving 25 is likely to have better consequences than saving one. Thus, failing to save the larger number would presumably be morally dubious also from a consequentialist perspective.

### Emotionally influenced behavior

Among the elements influencing moral behavior, emotions play an important role. Although, as for cognitive biases, people are usually unaware of the influence that emotions have on their behavior, several studies have shown that brain areas associated with emotion are involved in various decision-making tasks, including the formation of moral judgments.

A seminal study by Greene et al. (2001) has shown that emotions are usually sensitive to the means used for an action, while cognitive processes are sensitive to the consequences resulting from this action.

Other studies investigating the role of emotions in moral judgment showed that moral condemnation of an event (i.e., how wrong you think something is) is strongly influenced by the emotional state of the person evaluating it. Haidt and colleagues (Wheatley and Haidt, 2005; Schnall et al., 2008; Eskine et al., 2011) ran a series of studies which showed that induced disgust can yield harsher condemnations of a set of disgust-related moral violations such as incest.

Recently, we (Ugazio et al., 2012) have provided evidence that when a person judges a moral scenario, different emotional states will influence her choices in opposite ways. People who were induced to feel anger were more likely to judge a moral action in a permissive way compared to people in a neutral emotional state, and people induced to feel disgust were more likely to judge the same actions in a less permissive way.

The influence of emotional states on moral judgments and actions, in particular if the emotions stem from morally irrelevant factors of the situation, is morally problematic according to all the moral theories outlined in Section “How Ought We to Act?”. Consider consequentialism first and recall that from this perspective, the only aspects relevant to a moral evaluation are the outcomes of an action, decision, etc. In particular, the emotions of the agent are only relevant to the extent to which they are part of the overall utility affected by the outcomes. Hence, emotions are problematic if they influence an action or judgment such that it does not lead to the best possible outcome.

According to deontology, a morally right action or judgment is to be performed out of duty. Kant (1965/1785) famously declined that an action out of inclination fulfills this criterion. As emotions are regarded as inclinations of this sort, a judgment or action determined by an emotion cannot be morally right.

From the point of view of virtue ethics, the actions and judgments described in this section seem to be morally questionable because they do not seem to stem from virtuous practical reasoning. A virtuous person takes her passions into account in an adequate manner, yet she is presumably not dominated by their influence. Moreover, it might be the case that the actions and judgments described are morally dubious because they go against the virtue of temperance. However, the extent to which practical reasoning and temperance are non-virtuously counteracted will depend on the extent to which the action or judgment in question is influenced by the emotions.

Having given evidence for the claim that individuals often do not behave and judge in a morally sound way, we shall in the following section provide details on what we believe are the most important reasons for these failures.

## Why Do We Not Behave in Morally Decent Ways?

A first step toward a solution to the problem that people often do not behave in morally decent ways consists in analyzing the reasons and mechanisms of this behavior. Our hypothesis is that we do not behave in a way we ought to either because we have mistaken beliefs about what we ought to do or because we fail to carry out the right action despite our better knowledge.

For the first problem – we make mistakes in moral reasoning – a range of different causes can be given. The most obvious one is a lack of cognitive capacities. For instance, we suddenly find ourselves to be free-riders on a train because we simply forgot to validate our ticket. In this case, it is simply bad memory, lack of planning, distraction, or time pressure that led us to a moral transgression.

Inappropriate moral decision-making may also occur as a consequence of people’s ignorance of important information. Such ignorance then prevents them from drawing the correct conclusion how to act. For example, a consumer who wants to support fair working conditions may make a wrong decision because he is not aware that the company selling the product he chooses has recently been found guilty of sweatshop labor.

In addition, defective moral reasoning may be behind cognitive biases and phenomena such as the identifiable victim effect as described in the previous section. It seems to stem from a lack of reflection on the two scenarios, their comparison and moral evaluation, ultimately leading to the violation of the virtues of justice and fairness.

The second problem – despite knowing how we ought to act, we fail to carry out the right action – can be analyzed in a variety of ways. We will consider only a selection here.

Failure to act in a way that has been acknowledged of being the morally correct one may be due to personal weaknesses. The most prominent one is *akrasia*, sometimes also described as weakness of the will (cf. Kalis et al., 2008). We shall not distinguish between akrasia and weakness of will in this paper. A person is called akratic if she acts against her own standards or aims. Succumbing to some temptation, e.g., eating another portion of ice-cream despite your knowing you are thereby taking away someone else’s share, is usually regarded as an akratic action (cf. Austin, 1961).

The concept of akrasia depends heavily on the underlying idea of man. If we share Socrates’ view of a completely rational homo economicus, akrasia simply does not exist. Similarly, Aristotle and Aquinas have regarded akrasia as a result of defective practical reasoning whose result is a morally bad action (see also Hare, 1963; Davidson, 1970). However, if we believe that akrasia goes beyond fallacious reasoning, the difficult question arises of what akrasia actually is. Some have claimed that it is a conflict of competing forces, for instance, according to Augustine, between incompatible volitions. Others have described it as an instance of self-deception (Wolf, 1985; Schälike, 2004). In an Aristotelian vein, Beier (unpublished manuscript, see also Beier, 2010) argues that it is a result of underdeveloped virtues, that is, a defect in character building.

As far as we know, the philosophical concepts and theories concerning akrasia and related phenomena have not yet been linked to empirical research on defects of self-control, empathy, and self-involvement. Such an enterprise might, however, provide fruitful insights for both approaches. As the literature on behavioral and neuroscientific research is vast, we shall confine ourselves to a very brief review of evidence concerning *self-control* here.

An action out of self-control is generally defined as the choice of larger-later rewards over smaller-sooner ones (Siegel and Rachlin, 1995). Self-control has also been defined as the regulation of habits. From another perspective, self-control amounts to the control of emotional reactions (Ochsner and Gross, 2005). Both the second and the third approach regard self-control as a control of automatic reactions involving similar neural circuits. Neuroscientific research investigating the brain areas involved suggests that the dorsolateral prefrontal cortex (DLPFC) modulates the value signal encoded in the vmPFC which in turn drives choices and decisions (Hare et al., 2009). The DLPFC promotes task-relevant processing and eliminates irrelevant activities. Future research into DLPFC and its interactions with vmPFC and other brain regions may shed new light on how to analyze self-control and akrasia and how to influence those phenomena.

In sum, the philosophical conception of akrasia may be linked to a lack of self-control in the following way: relying on Beier, akrasia can be regarded as defective character building which essentially involves the development of self-control. This, in turn, will yield agents’ falling prey to morally irrelevant aspects of a situation, such as cognitive biases or emotional influences, which affect behavior and judgment. To illustrate, consider an example from the previous section: depending on their emotional states, subjects regarded moral transgressions more or less severe (Ugazio et al., 2012). That is, they could not separate their feelings from a consideration of a moral scenario which amounts to a defect of control over the emotions.

Another issue that hinders us from acting in morally decent ways may be certain *character traits*. For instance, fanatic religiosity sometimes turns people into murderers. Such traits are presumably the product of both genetic dispositions and their shaping through education and self-reflection.

A general reason for both morally fallacious reasoning and failure to carry out the action identified as the right one is the evolutionary background of human beings. Morality can be viewed as a product of the phylogenetic history of our species which has evolved in an environment different from the one we live in today. More precisely, it is commonly believed that reciprocity became a part of moral behavior because it enhanced the evolutionary fitness of reciprocating individuals (reciprocal altruism, cf. Trivers, 1971). Similarly, prosocial behavior within a group increased the reproductive abilities of its members in comparison to non- or anti-socially behaving groups (group selection, cf. Sober and Wilson, 1998). Likewise, altruistic behavior toward one’s own kin may increase the likelihood of spreading the shared genes (kin selection, cf. Hamilton, 1964).

To give some examples for evolutionary explanations of moral behavior, immediate and strong emotional reactions to a given situation probably evolved because they facilitate a quick reaction which in turn improved survival, for instance the fight-or-flight response to predators. The theory of kin selection can explain why humans evoke emotional reactions such as caring love toward their offspring and may favor them over foreigners: by helping the former and not the latter, their own genes are more likely to be passed on in the future. Likewise, we are now equipped with biases that automatically and unconsciously guide us in a way that helps to spread our genes. For instance, the identifiable victim effect increased the safety of the young in the agent’s close environment who shared genes with him with higher probability than did children further away. According to group selection, such biased behavior also improved the evolutionary fitness of one’s own group, as helping close-by group members rather than faraway out-group individuals would favor one’s own group and eventually the agent himself.

Related to this point, reasons for why we do not behave in morally decent ways can be regarded from a cultural perspective. On this view, morality can be seen as a relatively recent development, crystallized in laws and rules for social conduct. In this vein, the philosopher Nietzsche (1966, p. 228) has argued against moral systems such as Kantian, Christian, and Utilitarian ethics, criticizing that these codes of conduct are “detrimental to the higher men” while benefiting the “lowest.” From a similar perspective, morality may be seen as a fear of punishment which evolved originally and is exploited by legal systems. In this view, failures of morality arise whenever people do not experience enough fear of punishment. Presumably, the lack may come from the person or the situation.

Empirical research proves helpful to investigate and explain each of the problems mentioned, providing a basis on which we can search for solutions. We shall turn to this topic in the following section.

## Improving Moral Behavior

Having provided evidence (see section “People do not behave in a way they ought to”) that people often make inconsistent, if not mistaken, moral decisions and act accordingly, and having explored possible explanations for such irrational behavior (in the previous section), in this section we discuss possible means by which improving humans’ moral decision capacities, particularly via nudging, training, pharmacology, and lastly brain stimulation.

### Nudging

A nudge has been defined as an “aspect of the choice architecture that alters people’s behavior in a predictable way without forbidding any options” (Thaler and Sunstein, 2008, p. 6). Other than regulating, nudging does not eliminate possible courses of action. For example, a school canteen can increase pupils’ intake of vitamins by placing fruit salad or similar desserts in front of the chocolate cakes and sweets. This would be a nudge, whereas banning all alternatives to a healthy dessert would be a regulation. Nudging makes use of inclinations and biases, e.g., the fact that people tend to favor items displayed at eye level or often eat the portion they are served regardless of its size. Marketing strategies have benefited from these insights long ago, relying on long lasting research projects into consumer habits and psychology.

Nudging has mainly been investigated as a means to tackle population health issues, such as obesity and addiction to alcohol, nicotine, or other substances (Downs et al., 2009; Just and Payne, 2009; Zimmerman, 2009). However, it can be equally relied on in order to approach moral issues: it provides paternalistic institutions with strategies to succeed in guiding their clients, patients, or charges to the morally right decisions or actions (Thaler and Sunstein, 2003). For instance, given the assumption that organ donation is a morally praiseworthy action, a government can yield an increase in organ donors by making the donation of organs the default option of which you have to opt-out if you do not want to be a potential organ donor.

However, nudging in moral contexts raises a lot of issues. First, it is questionable whether a morally praiseworthy action loses its praiseworthiness if it had not been performed without the relevant nudge. This depends on whether an action is to be evaluated only on the basis of its results or also with regard to the states of mind of the agent. Second, as nudging itself seems morally neutral, the question arises how, taken in isolation, it could help us to improve moral decision-making and acting at all. Nudging may well be abused by the nudger for his personal interests. Third, the practice of nudging itself may be questioned on the ground of fear for autonomy and respect.

These and other questions will be discussed in Section “Should We Try to Improve, And Is It Possible?”. For now, we shall outline some further means and methods that might be useful for an improvement of moral practice.

### Training

Although already Aristotle suggested that sound judgment needs practice, there is little empirical research on direct training of moral decision-making. In as far as it is feasible to train cognitive and emotional functions and such training transfers to other domains it may also be conceivable to improve moral decision-making indirectly by training these functions. Working memory performance increases with training techniques such as an adaptive dual n-back task (e.g., Jaeggi et al., 2008), or an adaptive order-and-location memory task (e.g., Klingberg et al., 2005; Thorell et al., 2009). Working memory training transfers to other domains, including fluid intelligence (e.g., Jaeggi et al., 2008), attention (Thorell et al., 2009), and response inhibition, at least in children with ADHD (Klingberg et al., 2005). However, transfer appears to occur primarily in closely related domains (Li et al., 2008) and only in individuals in which initial training is successful (Jaeggi et al., 2011).

Response inhibition can be trained with go/no-go and flanker tasks (Thorell et al., 2009) whereas executive attention improves after training with a battery of anticipation and stimulus discrimination exercises (Rueda et al., 2005) but training effects seem to transfer less readily than with working memory training. Based on the hypothesis that utilitarian components of moral decision-making depend more on cognitive factors than deontological ones (Greene et al., 2001), one may speculate that training cognitive factors would improve specifically utilitarian components of moral decision-making. However, given that transfer appears to be limited to closely related domains, it is questionable whether moral behavior would benefit from such training.

Training of emotional factors can improve aspects of moral decision-making. For example, a Buddhist compassion-enhancing technique increases provision of help to another player in a virtual treasure hunt game (Leiberg et al., 2011). In the same game, the duration of compassion training correlates with helping particularly in situations in which the other player cannot reciprocate help. By contrast, compassion training does not affect giving money to others in a dictator game, where subjects decide how to split an amount of money assigned to them between a stranger and themselves (Leiberg et al., 2011). Taking these findings further, one may wish to investigate whether deontological components of moral decision-making are influenced more by emotion training than utilitarian components.

Through increasing effort-levels required for achieving reinforcement as well as exercises such as monitoring and improving posture, trying to improve mood states, and monitoring eating, self-control can be increased in humans and rats, respectively (reviewed in Strayhorn, 2002). Accordingly, it has been proposed that self-control acts like a muscle that can be trained or fatigued depending on experience (Baumeister et al., 1994). Insofar as self-control reflects a virtue, self-control training may be beneficial from a virtue ethics perspective.

### Education

Moral education has a long tradition and received consideration from all three philosophical theories introduced above (Althof and Berkowitz, 2006). It largely follows on from the (deontologically flavored) views of Piaget and Kohlberg and focuses primarily on the development of moral reasoning. By contrast, the related character education has a stronger grounding in virtue ethics and utilitarianism and aims to promote moral actions leading to good consequences in educated citizens (Althof and Berkowitz, 2006).

Within a Kohlbergian framework, interventions specifically designed to promote moral education are more effective than control interventions or the passage of time (Schlaefli et al., 1985; cf. King and Mayhew, 2002). Moreover, longer term (up to 12 weeks is optimal) interventions that focus on peer discussion of moral dilemmas, thereby leading to practice in moral problem solving, and interventions that focus on personality development and self-reflection are more effective than shorter-term interventions (≤3 weeks) and interventions that focus on academic content such as criminal justice, law, and social studies (effect sizes: 0.36–0.41 versus 0.09; Schlaefli et al., 1985). Treatment effects are more pronounced in older (≥24 years old) compared to younger (13–23 years old) subjects, although this may be partly due to selection bias (older subjects are more likely to be volunteers) or other methodological issues. Although the effect sizes of interventions are small to moderate, they lead to 4–5 years of natural growth compared to no intervention (Schlaefli et al., 1985), suggesting that education may be a promising avenue for future research.

### Pharmacological enhancement

The field of cognitive enhancement by pharmacological means has received attention in recent years (reviewed, e.g., in Jones et al., 2005; Illes and Sahakian, 2011) but the first empirical investigations have focused primarily on improving cognition as such, rather than on moral decision-making. Below, we review a few example studies with a more direct link to moral behavior. Before going further though, it is important to note a few caveats:

(1) It is not necessarily the case that more of a given pharmaceutical agent results in monotonic increases in function. Instead, at least some functions may require an intermediate level of the agent. Increases beyond that level result in decreases in the function. An example for this notion comes from working memory and dopamine (reviewed, e.g., in Cools and D’Esposito, 2011).
(2) Individual differences can moderate the relation of how pharmaceutical agents affect function. Such individual differences can be genetic or psychosocial. An example comes from the Taq1A DRD2 (dopamine D2 receptor) gene, where the presence of an allele (A1+) is associated with reduced dopamine receptor concentration, decreased neural responses to reward, but enhanced neural reward responses after delivery of a D2 receptor agonist compared to A1− subjects (Cohen et al., 2007). The endeavor of improving a given function may thus require tailoring agents and dosage to individuals.
(3) Improvements for one function may come at the expense of costs for another. For instance, improvements in social functions may come at a cost of reduced cognitive functions. Ethical questions become pertinent in this case in that one would have to argue why one function is ethically more important than another.
(4) Pharmaceutical agents administered systemically act in a sustained fashion over time but the relevant functions may be implemented in a temporally more phasic fashion. Moreover, the same pharmaceutical agent may have different functions at different time-scales (for dopamine e.g., Fiorillo et al., 2003; review in Schultz, 2007).

Intranasal administration of oxytocin (24 international units) increases trust in the trust game (Kosfeld et al., 2005). More specifically, the average initial amount passed by an investor to a trustee is 17% higher under oxytocin (45% of participants showing maximal trust) than under placebo (21%). Proposers’ offers are also enhanced by oxytocin in the ultimatum game (Zak et al., 2007). By contrast, non-social risk taking, trustworthiness of trustees (the amount returned by trustees) and amounts offered in the dictator game remain unaffected by oxytocin, excluding less specific effects on risk perception and prosociality more generally. Thus, oxytocin enhances an emotional aspect of moral behavior.

The administration of a selective serotonin reuptake inhibitor (30 mg Citalopram) increases the propensity with which people judge harming others as forbidden, if the inflicted harm is personal and emotionally salient (Crockett et al., 2010a). Moreover, it reduces the rejection of unfair offers in the ultimatum game (Crockett et al., 2010a; the rejection of unfair offers harms the proposer). Thus, serotonin may facilitate prosocial behavior or moral judgments more generally by enhancing aversion to harming others.

### tDCS/TMS

Transcranial direct current stimulation (tDCS) is a technique which allows for modulation of regional neural excitability by means of applications of weak currents. In short, neural activity (i.e., an action potential) is usually elicited when the membrane potential – usually −80 mV at rest – is lowered to about −50 mV via driving inputs through other neurons. Applying weak currents (usually 1 or 2 mA) over a cortical area can increase or decrease the resting membrane potential, depending on the position and polarity (anodal or cathodal) of the electrode. Thus, tDCS can lead to an increase or decrease of the excitability and spontaneous activity in the neural tissue under the electrode.

Transcranial magnetic stimulation (TMS) is a technique of non-invasive brain stimulation which uses magnetic impulses to generate weak currents in specific brain regions. So far, two types of TMS have been used, single pulse TMS and repetitive TMS (rTMS). The first type of stimulation affects neural excitability similarly to anodal tDCS, resulting in a depolarization of the neurons targeted by the magnetic impulses. Such depolarization then results in the generation of action potentials in the stimulated neurons. By contrast, rTMS lasts much longer than single pulse stimulation. Therefore rTMS can increase or decrease the resting membrane potential of the stimulated brain region, depending on the intensity and frequency of the stimulation and on the coil orientation (Fitzgerald et al., 2006).

Using both these techniques scholars have shown that it is possible to directly manipulate social and non-social behavior in several tasks including temporal discounting (Figner et al., 2010) and norm compliance (Ruff et al., in preparation). The latter study focused directly on moral behavior (i.e., complying with behavior prescribed by a norm). Other studies investigated processes which are related to moral behavior such as contributing to the enforcement of a fairness norm by costly punishing defectors, or mechanisms involved in shaping individuals’ impulsivity.

More specifically, Knoch et al. (2008) tested the role of DLPFC in punishing unfair behaviors. Measuring the altruistic punishments (Fehr and Gächter, 2002) responders inflicted to unfair proposers while playing an ultimatum game (Andreoni et al., 2003), the authors showed that reducing excitability by means of cathodal tDCS in the DLPFC led to a reduction of punishments, compared to participants with intact DLPFC excitability. Therefore the authors conclude that the DLPFC neural activity has a causal role in the willingness to punish fairness norm violators.

Furthermore, Figner et al. (2010) revealed a role of the LPFC for self-control in intertemporal choice behavior. Intertemporal choices require one to decide between receiving a smaller good (e.g., money or food, but also health benefits) in a closer future (usually immediately, but also in days or months) or a larger good in a distant future. Depending on the options an individual chooses it is then possible to measure its level of self-control: the more she prefers the distant-in-time option the higher her self-control level. Disrupting LPFC excitability by means of rTMS resulted in decreased self-control, as people chose more often the immediate smaller good over the alternative option.

Taken together these studies show that brain stimulation could influence two mechanisms strongly related to moral behavior, i.e., self-control and willingness to punish norm violators, as they are involved in social decisions where one is required to choose between a personal gain or benefiting the society (Elster, 1989; Fehr and Gächter, 2002; Fehr and Fischbacher, 2004; Crockett et al., 2010b).

Furthermore, the link between these two mechanisms and moral behavior is made more salient in a more recent study by Ruff et al. (in preparation). In this study we show that the LPFC is causally necessary to avoid altruistic punishment, inducing people to share fairly between oneself and another person when punishment for unfair behavior is allowed. More specifically, increased LPFC excitability (by means of anodal tDCS) resulted in more successful social interactions compared to decreased LPFC excitability (by means of cathodal tDCS) or natural LPFC excitability (sham stimulation). This study thus suggests that it is possible to improve moral behavior by increasing sensitivity to punishment threat, which is possibly achieved as a side effect of improving self-control.

Finally, in a more recent study, Tassy et al. (2012), examined the effects of disrupting the right PFC by means of rTMS on moral judgments expressed in the context of moral dilemmas where a person is called to judge if it is morally permissible to sacrifice a small number of people (usually one) to save the lives of many more (usually five). The evidence reported by these authors show that compared to controls with undisrupted right PFC activity, disruption leads to a higher likelihood of making utilitarian judgments.

## Should We Try to Improve, and is It Possible?

Relying on the evidence outlined so far, this final section discusses the question of whether we should make use of the knowledge gained from empirical research on human behavior and psychology in order to improve moral practice and/or decision-making. Even if we arrive at a positive answer to this question, however, it remains unclear, how this project ought to be carried out and whether, in turn, this is possible. We shall discuss the former question first and then turn to the question of implementation.

Whether we should strive for moral improvement depends on (a) whether we believe that it is something worth striving for, (b) whether, assumed that we think it is, we should strive for it, and (c) granted that we should, whether the methods and techniques outlined in this essay provide morally acceptable means for such a project.

(a) From a consequentialist perspective, moral improvement tends to be something worth striving for, granted that moral improvement is understood to yield overall better states of affairs. However, many people do not share such a consequentialist outlook. Moreover, morality does not seem to be something we can be passionate about and desire in itself (Wolf, 1982, p. 424). In a similar vein, Williams (1981) has argued that it is necessary for our existence to have some personal “projects,” i.e., action-guiding desires or aims which are distinct from the pure utilitarian pursuit of happiness or any other motivation derived from a moral theory.
(b) *Prima facie*, it seems odd not to strive for moral improvement if we acknowledge that it is worth striving for it. After all, it is widely assumed that if we consider something as morally good, we are motivated to act in a way to bring it about or at least not to act against it. Likewise, it is assumed that if we believe we are morally required to Φ, we are motivated to Φ (internalism). However, it is debatable whether this very assumption is correct. On the one hand, it is highly probable that we firmly believe in something’s being morally good or right and nevertheless do not act accordingly (externalism). Otherwise, the problem of akrasia would not even arise. On the other hand, even if a moral belief does motivate us in a certain way, this link itself may be questionable from a moral point of view. For instance, from a consequentialist perspective, it might be better if everybody acted upon certain rules laid out by some ethical framework, not upon their own moral convictions. A second point that can be made in this context is that, as a matter of fact, people generally strive for moral improvement or at least they claim to do so, i.e., they want to act in a more decent way, they want to become morally better individuals, they want the world to be a morally better place, etc. Three remarks shall be made about this: First, the folk notions of morally good individuals, actions, and states of affairs are vague and require clarification. Second, it is debatable whether people really do claim to strive for moral improvement and in which contexts and, again, what they understand by it. Third, it may be questioned whether their claim is appropriate, i.e., whether they are in fact concerned about moral improvements or only just say so. All these and other questions are worth pursuing in the future.
(c) An extended debate has arisen around this question for every single method we have outlined above (e.g., for the debate on enhancement: Douglas, 2008; Savulescu and Bostrom, 2009; Savulescu et al., 2011). Due to space limitations, we shall therefore only mention a few important arguments here.
  First, the mere possibility of moral improvement may count in favor of such a project, once it is acknowledged that moral improvement is desirable and ought to be aimed at. Furthermore, it may be viewed as an extension of methods that are already used for moral improvement at present, e.g., teaching, self-reflection, etc.
  Second, and in contrast to the position just sketched, it may be doubted that any of the methods and techniques provides an acceptable way to moral improvement at all. Several reasons may be given for this position. To begin with, one may be skeptical about whether any of the approaches outlined above can really yield actual moral improvement. After all, so far only small, primarily short-term and reversible effects have been achieved. Yet, although it seems plausible that there is a limit to improvement given the constraints of the human mind and body and that moral *perfection* cannot be achieved, it seems doubtful that it be not possible to improve at all. The empirical evidence we have reviewed above supports this notion.
  Also, it may be argued that the methods for improvement are not reliable because further research is required in order to allow for their responsible application. However, it may be replied from a consequentialist perspective that such risks can be accounted for by calculating the sum of all possible outcomes each multiplied with the probability of its occurrence. For some techniques such as nudging, no morally neutral default option is available: e.g., either a country’s citizens are organ donors by default or they are not, but each option invokes moral issues and there is no option outside of the moral realm.
  Third, a debunking argument in favor of applying the techniques and methods described could be established on the ground that all considerations speaking against such a project are merely products of a human *status quo* bias.

Much more could be said on each of the considerations described above. We assume that enough evidence suggests that attempts of moral improvement could be believed to be promising.

Let us now turn to the question of implementation: if we assume that we should try to achieve moral improvement, should such a project actually be carried out and if so, how? As the matter here is complex and partly speculative, we shall restrict ourselves to providing a brief sketch of two issues that are relevant to this debate.

To even start considering improving moral behavior, one has to first tackle the complex philosophical issue of identifying a standard for moral improvement. This might require defining an ultimate universally accepted moral code, or agreeing on a set of general moral rules, being these consequentialist rules or non-consequentialist ones. Such a standard would then have to be used to gear interventions used to improve moral behavior. Whether it is in principle possible to identify such a standard, however, is highly controversial. For one thing, moral relativists hold that moral standards are relative to a culture (Wong, 1984) and thus prescribe very different behaviors. Some, for instance, forbid abortion while others allow it. Improving moral behavior may thus be specific for every moral community sharing the same moral standards. More profoundly, one may be skeptical about whether it is in principle possible to achieve agreement on moral questions, given that current debates about moral issues reveal both intercultural and intracultural discrepancies. For instance, from a consequentialist perspective, it may be a moral improvement to increase the number of potential organ donors, but from some religious or deontological perspectives, this would be regarded as immoral.

Moreover, there is the danger of abuse by the agents or institutions in charge of implementing a process of moral improvement. Determining a prudent and trustworthy authority for this task may be extremely difficult if not impossible. Most people seem unwilling to entrust others with the care of their moral development.

Second, on a more practical stance, altering moral behavior may not yield the desired improvement effects or have counterproductive side effects. For instance, promoting trustfulness may result in exploitation of trustful agents, and increasing altruistic behavior may benefit unfairly selfish individuals who could easily take advantage of altruists. In addition, the danger of a moral “lock-in” is lurking: once a process of alleged moral improvement has begun, it may be irreversible, as the moral outlook produced by this process may prevent us from reviving lost values; mistakes may become uncorrectable.

In sum, the question of whether we should try to achieve moral improvement and whether this is possible raises a legion of extremely controversial questions. Note that the present paper itself does not mean to take a normative position on the issue of whether morality should be improved. The above points are merely meant to provide some leads for the debate.

## Conclusion

The aim of this paper was to investigate why individuals often fail to judge and act in a morally decent way and what one can do about it. Investigations on morally problematic and inconsistent behavior, dominated by, e.g., cognitive biases and emotional influences, have revealed two main clusters of reasons: first, agents reason in fallacious ways, and, second, in judging or acting, they fail to account for their moral convictions. These phenomena allow for several ways of improvement. For instance, nudging may facilitate actions in accordance with moral aims, training, and education may ameliorate agents’ capacities for moral reasoning, pharmacological enhancement and transcranial stimulation techniques may yield improvements of both moral reflection and capacity to act morally. However, impact and application spectrum of all these methods have not yet been thoroughly studied, as their development is still an on-going process. An answer to the question of whether they should be implemented not only depends on future research in this field but also requires careful philosophical consideration and societal debate. We believe that these endeavors are highly relevant for a possible improvement of moral practice and therefore for the future of humanity in general.

## Conflict of Interest Statement

The authors declare that the research was conducted in the absence of any commercial or financial relationships that could be construed as a potential conflict of interest.
